# Sequential CAR T-Cell Therapy After Autologous Stem Cell Transplantation for the Treatment of Relapsed/Refractory Intravascular Large B-Cell Lymphoma With Central Nervous System Involvement: A Case Report

**DOI:** 10.3389/fonc.2022.817969

**Published:** 2022-04-28

**Authors:** Wanying Liu, Chunrui Li, Yang Cao, Na Wang, Liang Huang, Zhen Shang, Jue Wang, Lifang Huang, Jinhuan Xu, Min Xiao, Yicheng Zhang, Jianfeng Zhou, Liting Chen, Yi Xiao

**Affiliations:** ^1^ Department of Hematology, Tongji Hospital, Tongji Medical College, Huazhong University of Science and Technology Wuhan, Hubei, China; ^2^ Immunotherapy Research Center for Hematologic Diseases of Hubei Province Wuhan, Hubei, China

**Keywords:** CAR T-cell therapy, IVLBCL, CNS involvement, B-cell lymphoma, intravascular

## Abstract

**Background:**

Intravascular large B-cell lymphoma (IVLBCL) is a rare, aggressive, large B-cell non-Hodgkin’s lymphoma. The prognosis of IVLBCL in patients with central nervous system recurrence after first-line chemotherapy treatment is extremely poor. Among immunotherapies, chimeric antigen receptor (CAR) T-cell immunotherapy has been recently found to be a highly effective treatment for B-cell lymphoma, especially for relapsed or refractory diffuse large B-cell lymphoma. However, no guidelines are available that provide a clear consensus regarding the management of patients with relapsed/refractory IVLBCL. Here, we report, for the first time, the use of autologous hematopoietic stem cell transplantation (ASCT) and CAR T-cell therapy in a patient with relapsed/refractory IVLBCL.

**Case Presentation:**

A 42‐year‐old woman was diagnosed with IVLBCL based on liver biopsy and developed central nervous system (CNS) progression. The patient received ASCT combined with murine monoclonal anti-CD19 and anti-CD22 CAR T-cell therapy. She achieved complete remission for 22 months so far with negative minimal residual disease and continues to be followed up.

**Conclusion:**

ASCT combined with CAR T-cell therapy was the best choice for treatment of relapsed/refractory IVLBCL, as it allowed the achievement of a lasting complete remission.

## Introduction

Intravascular large B-cell lymphoma (IVLBCL) is a rare, aggressive, large B-cell non‐Hodgkin’s lymphoma in which neoplastic B cells proliferate selectively within the lumina of small- and medium-sized vessels. IVLBCL is a rare subtype of diffuse large B-cell lymphoma (DLBCL), with an estimated incidence <1 per million. Recently, IVLBCL has been categorized as a subtype of extranodal large B-cell lymphoma according to the World Health Organization (WHO) classification criteria (1). IVLBCL shows a high incidence of neurological symptoms, and commonly involved sites include bone marrow, spleen, skin, liver, adrenal gland, brain, lung, bone, kidney, and uterus (2).

IVLBCL is an aggressive disease with a poor outcome, and clinical signs and symptoms are nonspecific; therefore, the diagnosis is commonly missed. In fact, many cases were confirmed in post-mortem evaluation. Owing to the rarity of IVLBCL, treatment recommendations are largely based on retrospective studies and case reports. The combination of rituximab and cyclophosphamide, vincristine, doxorubicin, and prednisolone (R-CHOP) is widely used as first-line therapy, but the 2-year progression-free survival (PFS) and overall survival (OS) rates are only 56% and 66%, respectively (3). In a recent study, CNS involvement was identified as a risk factor for poor outcomes (4). In patients without CNS involvement at diagnosis, the risk of CNS recurrence at 3 years, both with and without CNS prophylaxis, was 25% (5). The prognosis of the four IVLBCL patients who developed CNS involvement during or after R-CHOP chemotherapy was extremely poor, as three patients died from disease progression (6). Therefore, more effective treatment strategies should be developed to improve the outcomes of patients with CNS involvement. In the phase II PRIMEUR-IVL trial, the 2-year PFS of patients with previously untreated IVLBCL who received R-CHOP with high-dose methotrexate and intrathecal chemotherapy as CNS-oriented therapy was 76% (7). More intensive therapies, such as autologous hematopoietic stem cell transplantation (ASCT), have been pursued to overcome this problem. ASCT has been reported to result in prolonged survival for patients, with 2-year PFS of 81% (8). As one of the most promising fields of cancer treatment, chimeric antigen receptor (CAR) T-cell immunotherapy has already shown good outcomes in patients with refractory and relapsed DLBCL and B-cell ALL (9). However, there is no research on the application of CAR T-cell immunotherapeutic strategies to IVLBCL. Here, we report the successful use of CD19/22 CAR T-cell cocktail immunotherapy combined with ASCT in IVLBCL. To the best of our knowledge, this is the first case of CAR T-cell therapy applied to IVLBCL. This therapeutic regimen may improve the long-term outcomes of relapsed/refractory (r/r) IVLBCL.

## Case

**Figure 1:**
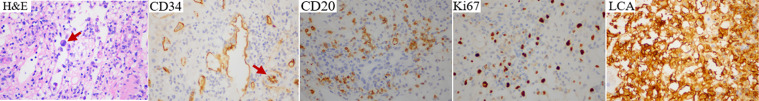
Hematoxylin and eosin (H&E) and immunohistochemistry staining of relapsed/refractory intravascular large B-cell lymphoma.

**Figure 2:**
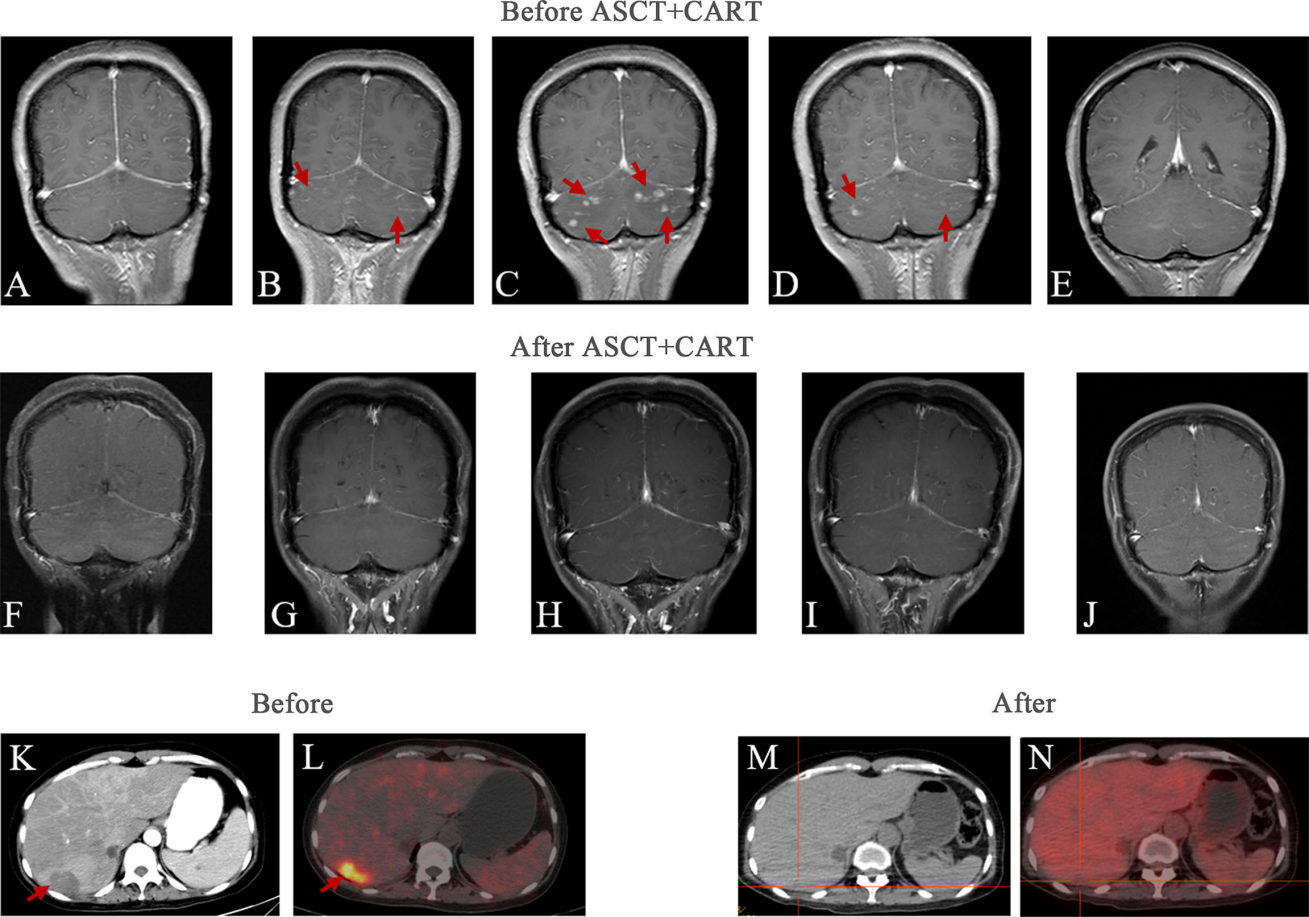
Head and liver imaging results of relapsed/refractory intravascular large B-cell lymphoma. Brain MRI images: **(A)** First time headache symptoms appeared at the initial diagnosis. **(B)**The intracranial infiltration was diagnosed. **(C)** 1 month after intrathecal chemotherapy. **(D)** 1 month after two courses of the adjusted treatment regimen MTX combined with Ara-C therapy. **(E)** 1 month before ASCT and CAR T-cell therapy. **(F)** 3 months after ASCT and CAR T-cell therapy. **(G)** 6 months after ASCT and CAR T-cell therapy. **(H)** 9 months after ASCT and CAR T-cell therapy. **(I)** 12 months after ASCT and CAR T-cell therapy. **(J)** 22 months after ASCT and CAR T-cell therapy. Abdominal images: **(K)** Abdominal CT at initial diagnosis. **(L)** PET-CT at initial diagnosis. **(M)** The most recent Abdominal CT after therapy. **(N)** The most recent PET/CT after therapy.

A 42-year-old woman presented to a local hospital on January 1, 2019, for a persistent fever of unknown reason that had been occurring for 1 month accompanied by headache. Laboratory test results are as follows: hemoglobin 48g/L, platelet count 54×10^9^/L, ALT 90.6U/L, AST 87.2U/L, total bilirubin 32.8umol/L, LDH 636 U/L, ferritin>2000ng/ml. Bone marrow cytology showed reactive bone marrow images, and hemophagocytic cells were occasionally observed. Abdominal computed tomography (CT) (**Figure 2K**) revealed. multiple space-occupying lesions in the liver, the largest is about 3.7cm in diameter. In addition, there was a hepatic cyst with a diameter of about 1.6 cm in the liver. Positron emission tomography-computed tomography (PET-CT) revealed multiple density shadows of abnormally high uptake of fluorodeoxyglucose (FDG) in the liver, the largest being about 3.7×2.9cm, the early maximum standard uptake value (SUV _max_) of the liver was 6.3, and the delayed SUV _max_ was 8.2 (**Figure 2L**). A round low-density foci with no FDG uptake can be seen in the right lobe of the liver, about 1.7 × 1.5 cm in size. Abnormally increased FDG uptake in the upper abdominal cavity, retroperitoneum, and para-vascular lymph nodes with a maximum lymph node size of 1.5 × 1.1 cm and a maximum SUV of 3.1. On January 14, 2019, the patient suddenly experienced headache, nausea, vomiting, and started speaking discontinuously and illogically. She gradually developed irritability, delirium, and coma. Enhanced magnetic resonance imaging (MRI) (**Figure 2A**) of the head just revealed a few ischemic degeneration foci in the brain. As the patient has chronic viral hepatitis B, hepatocellular carcinoma was the main differential diagnosis. On January 25, 2019, ultrasound-guided percutaneous liver biopsy was performed under the suspicion of an unusual malignant tumor at the local hospital. Immunohistochemical staining revealed that the tumor cells were positive for CD20, CD34, and LCA and negative for CD3, CD30, CD68, MPO, CKpan, Syn, CgA, and CK8/18(**Figure 1**). The Ki-67 proliferation index was estimated to be 80%. She has no family history of hematological malignancies. In summary, she was diagnosed with IVLBCL at stage IVB according to Ann Arbor staging and had a hemophagocytic syndrome. IPI was 3 and ECOG performance status was 1.

The patient was started on induction chemotherapy with two courses of R-DEP (rituximab600mgd0, doxorubicin20mg d1, etoposide100mg d1, and prednisolone80mg d1-5) for hemophagocytic syndrome and two courses of R-CHOP (rituximab600mgd0, doxorubicin40mgd1, cyclophosphamide1.1gd1, vindesine4mgd1 and prednisolone100mg d1-5), then she attained partial remission by PET-CT. Subsequently, she experienced paroxysmal headache during the third R-CHOP chemotherapy regimen. Combined with the history of the disease, she was considered to have CNS progression, which was identified using head MRI (**Figure 2B**). The head MRI showed multiple short-line and nodular enhancements in the cerebellum, the cerebral meninges and in the brain parenchyma at the junction of the right temporal-occipital lobe. Then she was administered CNS-directed chemotherapy including two courses of methotrexate (MTX)-based combination regimens (intrathecal injection MTX 10 mg, Ara-C 50 mg, dexamethasone 5 mg) and high-dose MTX therapy (rituximab 600 mg d0, MTX 5.4 g d1), but they did not work as re-examination of the head MRI (**Figure 2C**) after 1 month showed intracranial progress. The patient then received two courses of the adjusted treatment regimen MTX combined with Ara-C therapy (MTX5.4g d1, Ara-C 1.5g q12h d2-d3). Head MRI (**Figure 2D**) shows improvement compared with previous one.

**Figure 3:**
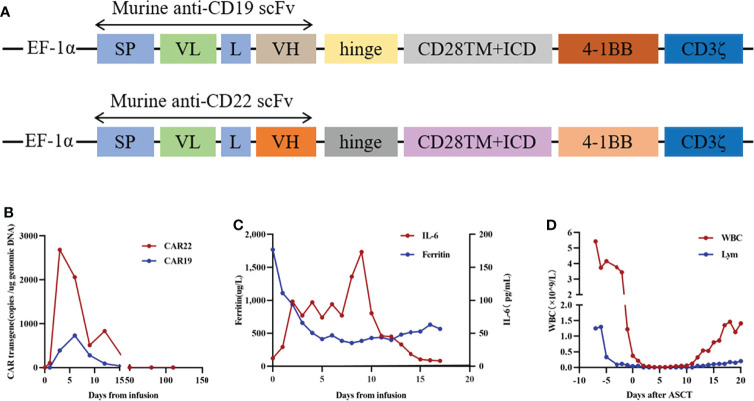
The protocol for the murine monoclonal anti-CD19 and anti-CD22 CAR T cell “*cocktail*” followed by ASCT and therapeutic response. **(A)** Schematic diagram of murine CAR19 and CAR22 CAR vectors. SP, signal peptide; VH, variable H chain; L, linker; VL, variable L chain. **(B)** Timeline of murine CAR22 and CAR19 transgene copy numbers. **(C)** Dynamic changes in IL-6 and ferritin after CAR T cell infusion. **(D)** Dynamic WBC numbers and lymphocyte numbers before and after CAR T cell therapy.

**Figure 4:**
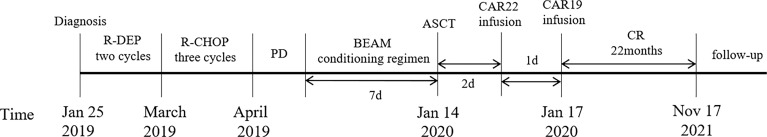
Timeline of disease status and corresponding treatment regimens.

To prevent the progression of the disease, she came to our hospital for CAR T-cell therapy, and we decided to perform ASCT combined with CAR T-cell therapy. The trial was approved by the Institutional Review Board of Tongji Hospital, Tongji Medical College, Huazhong University of Science and Technology, and the study was registered with the Chinese Clinical Trial Registry (ChiCTR-OPN-16009847). Informed consent was obtained by the patient and her family according to the Declaration of Helsinki. Before the conditioning regimen, the patient received two separate apheresis procedures, including granulocyte colony-stimulating factor (G-CSF)-stimulated autologous hematopoietic stem cell (HSC) collection and peripheral blood mononuclear cell (PBMC) apheresis for CAR T-cell manufacturing. CAR T-cell manufacturing-related quality control and analysis were completed by Wuhan Bio-Raid Biotechnology Co., Ltd., as previously described (10). Due to the patient’s refractory characteristics, a cycle of standard salvage treatment with rituximab, dexamethasone, cytarabine, and cisplatin (R-DHAP) was initiated in the mid-November 2019. Fourteen days later, autologous stem cells were harvested (3.08×10^6^ cells/kg). The head MRI 1 month before ASCT and CAR19/22 T-cell cocktail therapy treatment is shown in **Figure 2E**. The patient was administered a standard dose of the BEAM (carmustine, etoposide, cytarabine, melphalan) regimen one week before ASCT. On January 14, 2020 (day 0), the patient underwent ASCT (CD34 3.31×10^6^ cells/kg). Autologous CD22-targeted CAR T-cells (CAR22) (2.7×10^6^ cells/kg) were infused on day +2, followed by CD19-targeted CAR T-cells (CAR19) (4.3×10^6^ cells/kg) on day +3. The structure is as shown in **Figure 3A**. Both CAR19 and CAR22 transgene copy numbers were determined by droplet digital polymerase chain reaction (ddPCR) (**Figure 3B**). After sequential infusion of anti-CD19 and anti-CD22 CAR T-cells, grade 1 cytokine release syndrome (CRS) was observed, and interleukin-6 and ferritin levels increased slightly and transiently (**Figure 3C**). Since the patient had hepatitis B virus, we continued to monitor her hepatitis B virus titers continuously. Before and after CAR T-cell therapy, both were <1.0×10^2^ IU/mL, and there was no hepatitis B virus activation after treatment. The dynamic changes in white blood cells and lymphocytes after CAR T-cell therapy are depicted in **Figure 3D**. The leukocytes were engrafted at 2 weeks, and bone marrow hematopoiesis recovered in about 20 days. B cell recovered in about six months. CAR T-cell number and the copies of CAR22 and CAR19 increased dramatically and reached a peak in the first week. Follow-up after ASCT and CAR19/22 T-cell cocktail therapy, the patient’s cerebrospinal fluid leukemia/lymphoma minimal residual disease immunophenotyping test continued to be negative every 3 months. Sustained complete remission on head MRI at 3 (**Figure 2F**), 6 (**Figure 2G**), 9 (**Figure 2H**), and 12 (**Figure 2I**) months after ASCT and CAR T-cell therapy. Twenty-two months after ASCT and CAR19/22 T-cell cocktail therapy, the patient was still disease-free, and the head MRI image is shown in **Figure 2J**. The most recent FDG-PET/CT on November 17, 2021 was unable to detect any significant disease, and the infiltration of tumor cells into the liver continued to vanished (**Figure 2N**). Liver CT showed no new lesions (**Figure 2M**). No tumor cells were found in the cerebrospinal fluid. The timeline of the clinical treatment and disease status were shown in **Figure 4**.

When it comes to her thoughts on the disease and treatment, she expressed her great gratitude to our hospital and her husband. Despite her reluctance to face the diagnosis of lymphoma, the encouragement and positivity of her family (especially her husband) fueled her courage and strength to overcome the disease. She felt hopeless and helpless when she learned of her central involvement, poor prognosis and limited treatment options. When she and her husband learned of the clinical trials being conducted at our hospital, her desire to survive was rekindled, and she volunteered to participate in the clinical trials. Currently, the patient is working again, grateful, optimistic, healthy, and is very satisfied with her situation.

## Discussion and Conclusion

IVLBCL is a rare, aggressive disease with a dismal prognosis, especially in patients with relapsed or progressive disease after first-line treatment. CNS recurrence is a serious complication in patients with IVLBCL, and optimal treatment strategies for CNS involvement should be established to further improve clinical outcomes in the rituximab era. However, there is still no consensus concerning this issue. Early diagnosis and treatment are of great significance for improving the prognosis of IVLBCL patients. Significantly, PET-CT is a powerful tool for the early diagnosis of IVLBCL by identifying the indicated sites for biopsy relying on FDG uptake. Ultrasonography-guided percutaneous liver biopsy was performed in our patient because of the abnormally high FDG uptake in the liver. In this patient, detection of CNS involvement was diagnosed based on neurological symptoms, along with corroborating findings on imaging modalities.

Although rituximab has improved the outcome of patients with IVLBCL, more treatment strategies are still needed to deal with r/r IVLBCL. In a single institution in Taiwan containing ten patients with IVLBCL, it was shown that patients might achieve durable remission after rituximab-based chemotherapy (2). In a recent study, the outcomes of four patients who had CNS relapse after R-CHOP chemotherapy were extremely poor, as three patients died from disease progression (6). This suggests the need for more effective consolidation treatments for patients at high risk for treatment failure. Rituximab-containing chemotherapies combined with effective CNS prophylaxis may reduce the risk of CNS recurrence. A retrospective study found that 8 of 10 IVLBCL patients who received CNS treatment for CNS involvement at diagnosis survived without recurrence until the end of the study, indicating the promising potential for CNS prophylaxis and treatment (5). However, in a recent retrospective study on patients with IVLBCL with CNS involvement, the application of blood-brain barrier-penetrating chemotherapy did not improve outcomes (4). Alternatively, the up-front ASCT during the first complete remission (CR) may be a useful treatment option for patients with IVLBCL. Only one of six patients undergoing salvage ASCT after relapse survived, while two patients receiving upfront ASCT, including one patient with CNS involvement, were still alive without relapse at the end of the study period. This showed better outcomes for upfront ASCT (6). A registry-based study also suggested a favorable outcome of upfront ASCT in 11 patients with IVLBCL. Two-year PFS and OS rates were 81% and 91%, respectively (8). ASCT alone was also an option. Although studies have shown that early ASCT may improve the results of standard induction chemoimmunotherapy in these rare high-risk IVLBCL patients, the treatment options for recurrence after ASCT treatment are limited for patients with r/r IVLBCL. Therefore, there is an urgent need for new treatment methods to further stabilize the efficacy of ASCT and prevent disease recurrence, especially in patients with IVLBCL with CNS involvement.

CAR T-cell immunotherapy has already shown good outcomes in patients with r/r DLBCL and B-cell ALL (9). Recently, combined CD19/CD22 chimeric antigen receptor T cell cocktail therapy with ASCT in patients with relapsed/refractory aggressive B cell lymphomas has shown encouraging results (10). We pioneered the use of CAR T-cell immunotherapy with ASCT to treat IVLBCL and achieved remarkable results. Our case shows a long-term response of r/r IVLBCL patients to ASCT sequential CAR T-cell infusion treatment, and CRS is controllable. The possible reasons are as follows. First, an enhanced conditioning regimen and ASCT before CAR T-cell immunotherapy can minimize the tumor burden, reduce the immunosuppressive tumor microenvironment, and deeply deplete lymphocytes that inhibit the function of CAR-T cells (11). Second, myeloablative conditioning may help open the blood-brain barrier and facilitate the entry of CAR T-cells to the CNS to exert anti-tumor effects. Finally, for this type of lymphoma characterized by the proliferation of neoplastic cells in the lumen of small blood vessels, CAR T-cells may better reach the tumor site through blood circulation, which further improves the curative effect of CAR T-cell immunotherapy.

Antigen escape and downregulation have emerged as major issues affecting the durability of CAR T-cell therapy. Early-phase clinical trials demonstrate robust efficacy in several CD19 CAR T-cell products. Recently, CAR22 T cells were shown to induce durable remission in patients who were resistant to prior CD19-directed immunotherapies (12). Subsequently, a CAR that simultaneously targets both CD19 and CD22 was developed, which could prove more effective at inducing remission and could be less susceptible to relapse associated with antigen escape (13). Our team conducted a clinical trial to test this CD19–CD22-multispecific CAR and showed that the infusion of CAR19/22 T-cell cocktail is efficient and safe for patients with r/r B-cell malignancies (9). Dual CD19 and CD22 targeting is a promising approach for reducing antigen-loss relapse in CD19/CD22-directed therapy. To this end, we first administered sequential CAR T-cell infusions targeting dual CD19 and CD22 in IVLBCL patients to prevent antigen escape. In view of CNS infiltration, which is a sign of poor prognosis, and in order to further enhance the curative effect, we adopted a treatment strategy of ASCT combined with CAR T-cell therapy, although this is not a standard approach. The patient achieved CR and had no evidence of disease recurrence after 22 months of follow-up.

In conclusion, this is the first time that CD19/22 sequential CAR T-cell combined with ASCT therapy has been used as a novel therapy in IVLBCL. This one case report where ASCT and CD19/22 CART resulted in long-term remission and was safe, suggests that this approach may be promising and should be considered for further study. However, there are some limitations in our present study. Clinical trials in a wider population are difficult due to the rarity of ILBCL cases. It still requires more patients and clinical trials to verify. Our successful case of using sequential CD19/22 CAR T-cell immunotherapy followed by ASCT provides new treatment options for r/r IVLBCL patients. This approach merits further study in CNS-involved IVLBCL patients.

## Data Availability Statement

The original contributions presented in the study are included in the article/supplementary material. Further inquiries can be directed to the corresponding authors.

## Ethics Statement

The studies involving human participants were reviewed and approved by Medical Ethics Committee of Tongji Hospital, Tongji Medical College, Huazhong University of Science and Technology (TJ-IRB20160310). The patients/participants provided their written informed consent to participate in this study.

## Author Contributions

WL and CL analyzed the data and wrote the manuscript. LC and YX revised the manuscript. YC, LH, and MX performed the experiments. NW, ZS, JW, LFH, and JX took care of the patient. YZ and JZ provided clinical information. JZ, LC, and YX directed the research. All authors contributed to the article and approved the submitted version.

## Funding

This work was supported in part by the National Natural Science Foundation of China (No. 81830008 to JZ, No. 81700160 to Liting Chen, No. 81873444 and No. 82070213 to YX) and the Hubei Provincial Science and Technology Key Research Program (No. 2020BCB021).

## Conflict of Interest

The authors declare that the research was conducted in the absence of any commercial or financial relationships that could be construed as a potential conflict of interest.

## Publisher’s Note

All claims expressed in this article are solely those of the authors and do not necessarily represent those of their affiliated organizations, or those of the publisher, the editors and the reviewers. Any product that may be evaluated in this article, or claim that may be made by its manufacturer, is not guaranteed or endorsed by the publisher.
